# When Affection Becomes Risk: Human–Dog Interactions Associated with Bite and Scratch Injuries in a Survey of Dog Owners’ Knowledge, Attitudes, and Practices in Thailand

**DOI:** 10.3390/ani16121809

**Published:** 2026-06-11

**Authors:** Tuempong Wongtawan, Prapawee Sungkatavat, Onphirul Yurachai, Natalie Waran, Worakan Boonhoh

**Affiliations:** 1 Akkhraratchakumari Veterinary College, Walailak University, Nakhon Si Thammarat 80160, Thailand; tuempong.wo@mail.wu.ac.th (T.W.);; 2 One Health Research Center, Walailak University, Nakhon Si Thammarat 80160, Thailand; 3 Pet Mental and Behavior Clinic, Animal Hospital, Walailak University, Nakhon Si Thammarat 80160, Thailand; 4 Division of General Communicable Disease, Department of Disease Control, Ministry of Public Health, Nonthaburi 11000, Thailand; 5 Navigate Welfare, Napier 4182, Hawke’s Bay, New Zealand; 6 Center of Excellence in Innovation on Essential Oil and Bioactive Compounds, Walailak University, Nakhon Si Thammarat 80160, Thailand

**Keywords:** canine, misconceptions, owner-directed aggression, pets, stranger-directed aggression

## Abstract

Owning a pet dog provides significant mental and physical health benefits for humans. However, undesirable canine behaviors may result in bite or scratch injuries, leading to infection risks, psychological consequences, and potential dog abandonment. This study hypothesized that specific human–dog interactions are associated with unwanted canine behaviors and may increase the risk of injury in humans. The objectives were to determine the prevalence of dog-related bite and scratch injuries; assess dog owners’ knowledge, attitudes, and practices; and evaluate their associations with specific interaction patterns. Data were collected through an online survey of dog owners in Thailand. Most respondents were young women with a bachelor’s degree, residing in Central Thailand, and owning a single dog. Interactions associated with increased injury occurrence included grooming; administering medication; removing food or toys; interrupting dog fights; and close physical contact including petting, hugging, and kissing. Injuries in strangers were most commonly triggered by entry into the dog’s territory. Younger owners (18–34 years) were at increased risk, particularly during grooming and frequent petting. General awareness of rabies, bacterial infection, and wound management was adequate. However, there were still misconceptions about dog behavior. These findings highlight key risk factors and support targeted educational interventions to reduce injuries and improve animal welfare.

## Simple Summary

This study explored which interactions between humans and pet dogs may lead to bite or scratch injuries, using an online survey of dog owners in Thailand. Most participants were young women who owned one dog. Injuries most commonly happened during close or stressful interactions, such as grooming, giving medication, taking away food or toys, stopping dog fights, or excessive close physical contact like hugging and kissing. Encounters with strangers often led to injury when a person entered the dog’s territory. Younger owners were more likely to be injured, especially during grooming and frequent petting. Although many owners were aware of rabies and basic wound management, some lacked accurate understanding of dog behavior and risk situations. Misconceptions about how dogs react to certain human actions were common. Overall, this study highlights that specific human–dog interactions can increase the risk of injury.

## 1. Introduction

Pet dogs have become integral companions in human life, with ownership providing multiple health benefits. Physically, dog ownership has been associated with a reduced risk of cardiovascular disease and obesity [1,2]. In terms of mental health, it has been shown to alleviate depression, decrease loneliness, and promote social interaction [3,4].

However, some dogs may exhibit undesirable behaviors such as aggression, over reactivity, and destructiveness, which can strain or even threaten the human–dog bond [5,6]. Such behaviors can arise from a variety of factors, including genetics, environmental influences, and the nature of human–dog interactions [7,8,9,10]. In particular, injuries caused by dogs, including bites and scratches, pose direct risks to humans through tissue trauma, potential pathogen transmission, and psychological impacts, especially in children [11,12,13,14]. Although bites and scratches may arise through different behavioral mechanisms and may vary in severity, these behaviors are frequently linked to aggression [15].

Various human–dog interactions have the potential to provoke canine aggression-related behaviors, particularly those that involve frightening, threatening, causing pain, or creating discomfort for the dog [16]. Fear is one of the most frequent underlying triggers, arising when dogs are intimidated by their owners; confronted by strangers; or exposed to uncomfortable contexts such as grooming, punishment, or veterinary visits [17,18]. Fear-related aggression is considered a defensive reaction, occurring when a dog perceives a threat or experiences fear, and manifests through behaviors such as growling, barking, snarling, biting, or scratching in an effort to protect itself or escape the situation [19]. Canine behaviors associated with biting or scratching are often context-dependent and may represent communication attempts, defensive responses, or efforts to avoid perceived threats rather than indiscriminate aggression. In addition to fear, defensive-related aggression in dogs may also arise from their instinctive drive to protect territory (known as territorial aggression) or valuable resources (known as resource guarding) against perceived intruders or threats [15,20].

Incidents of injury-associated behaviors in Thailand are frequently reported in the media and involve both owned and stray dogs. However, these events remain poorly characterized in the scientific literature, particularly in relation to owner behavior, interaction contexts, and preventive practices. The most recent published estimate of dog-bite incidence in Thailand dates back more than three decades, reporting that dog bites accounted for 5.3% of injuries presenting to emergency departments in hospitals in the Bangkok region [11]. Dog bites and scratches not only cause physical injury but also serve as important routes of transmission for zoonotic pathogens, including bacterial and viral agents [12,21,22]. Rabies, a universally fatal viral disease, represents one of the most significant zoonoses transmitted by dogs and other mammals and remains a major public health concern in Thailand. A recent study estimated that 47% of dogs in Thailand are at risk of rabies virus infection [22,23]. Most previous studies have focused on clinical presentations, hospital-based surveillance, or aggression case reports, often emphasizing severe bite outcomes or veterinary behavioral referrals [11,15,16]. Consequently, relatively little is known about the broader spectrum of owner-reported dog-related injuries occurring during every day human–dog interactions.

In this study, we hypothesized that particular patterns of human–dog interaction may be associated with aggression-related behavior and with the occurrence of human bite and scratch injuries. The objectives of this study were to estimate the prevalence of dog-related biting and scratching injuries in humans; to evaluate the knowledge, attitudes; and practices of dog owners, and to explore their relationships with particular patterns of human–dog interaction. Unlike hospital-based studies, this survey captures both medically attended and non-medically attended injury events, thereby providing broader insight into the circumstances associated with dog-related injuries and opportunities for prevention. To our knowledge, this represents one of the first national studies in Thailand to integrate epidemiological, behavioral, and educational dimensions of dog bite and scratch injuries.

## 2. Materials and Methods

### 2.1. Ethical Approval

The study was approved by the Human Research Ethics Committee of Walailak University (WUEC-25-051-01). This study was conducted using an online questionnaire. All participants were required to read the research proposal and consent form, which provided agreement and consent to the stated conditions before completing the questionnaire. All responses the respondents provided for this study were completely confidential, and they cannot be identified by name or any other information that could be used to infer their identity.

### 2.2. Survey

The present research was part of an online survey on bite and scratch injuries by pets in Thailand. The questionnaire was administered online using Google Forms (Google LLC, California, CA, USA). Thai dog owners were invited to participate in the study through advertising online by social online platforms such as Facebook, Instagram, Line application, and also pet-related websites. For onsite advertising, we posted posters with the QR code to many animal hospitals around Thailand, which allows the respondent to use their mobile phone to scan the QR code and answer the online questionnaire. The survey took approximately 15–20 min to complete. Data collection was conducted over a four-month period, from March to June 2025. The target population of this online national survey were Thai citizens who were at least 18 years old, owned a dog(s), lived in Thailand, and may have experience with pet bite and/or scratch injury.

### 2.3. Questionnaire

The questionnaire consisted of sections on demographic variables and details of dog-related biting and scratching injuries in humans. Demographic variables included owner and dog characteristics such as owner gender, age, education level, and living region, as well as the number of dogs in the household, vaccination history, and presence of chronic diseases in dogs.

The injury section collected information on the incidence of bites and scratches involving owners and strangers, frequency of such events, specific human–dog interactions, owners’ attitudes on potential triggers, and owners’ knowledge on dog bite and scratch relation and prevention. The questions are shown in Supplementary Table S1.

The questionnaire collected owner-reported information regarding dog bite and scratch injuries involving both owners and strangers. In this study, bite and scratch events were assessed based on respondents’ own interpretations and experiences of injury occurrence. The survey was designed to capture dog-related injury experiences broadly and did not classify bite severity; distinguish between skin-breaking and non-skin-breaking bites; or specifically differentiate behaviors such as inhibited biting, pressure-only contact, or mouthing. Consequently, reported events reflect owner-perceived bite and scratch incidents rather than standardized clinical or behavioral diagnoses.

### 2.4. Statistical Analysis

According to data from the Department of Livestock Development, there were 2,064,876 registered pet dogs in Thailand. Based on Yamane’s formula [24], the minimum required sample size was 400 completed questionnaires.

Data analysis was performed using Jamovi version 2.5 (https://www.jamovi.org). Chi-square tests were applied to examine associations between variables and to assess statistical differences among groups. A *p*-value of ≤0.05 was considered statistically significant. Spatial distribution patterns of dog bite and scratch incidents were generated using ArcGIS online version (ESRI Inc., Redlands, CA, USA).

## 3. Results

### 3.1. Demographic Characteristics of the Owners and Dogs

A total of 1053 dog owners participated in this study, representing all five regions of Thailand (central, northeastern, eastern, northern, and southern regions). Respondents were drawn from nearly all provinces (72 of 77; 93.50%), with the exception of Trat, Satun, Kalasin, Nakhon Phanom, and Nong Bua Lamphu. The demographic characteristics of both the dogs and their owners are presented in Table 1.

The majority of respondents were female (77.69%, *n* = 818), aged between 18 and 30 years (32.95%, *n* = 347), held a bachelor’s degree (57.46%, *n* = 605), and resided in the Central region of Thailand (41.03%, *n* = 432). Most owners reported having a single dog in the household (41.31%, *n* = 435). With regard to canine health, the majority of dogs received annual rabies vaccination (79.68%, *n* = 839) and did not suffer from chronic diseases (62.96%, *n* = 663) (Table 1).

### 3.2. Occurrence of Dog Biting and/or Scratching Human

Of the 1053 respondents, 20.60% (*n* = 217) reported bite injuries and 10.06% (*n* = 106) reported scratch injuries caused by their own dogs, and 14.81% (*n* = 156) of the owners reported both outcomes. When combining the analyses of bite and scratch injuries, 45.48% (*n* = 479) of the respondents reported overall dog-related injury occurrence. Among these 479 participants, the majority reported injury incidents occurring every three months or less frequently (70.15%, *n* = 336), followed by monthly incidents (11.69%, *n* = 56) and weekly incidents (10.43%, *n* = 50). Notably, 7.72% (*n* = 37) experienced biting or scratching on a daily basis.

Among the 1053 respondents, 16.61% (*n* = 175) of respondents reported that their dogs had bitten other adults, 2.75% (*n* = 29) of pet dogs were reported to have bitten stranger children, and 6.93% *(n* = 73) reported incidents involving both adults and children, yielding an overall occurrence of 26.30% (*n* = 277) of pet dogs that had bitten strangers.

**Figure 1:**
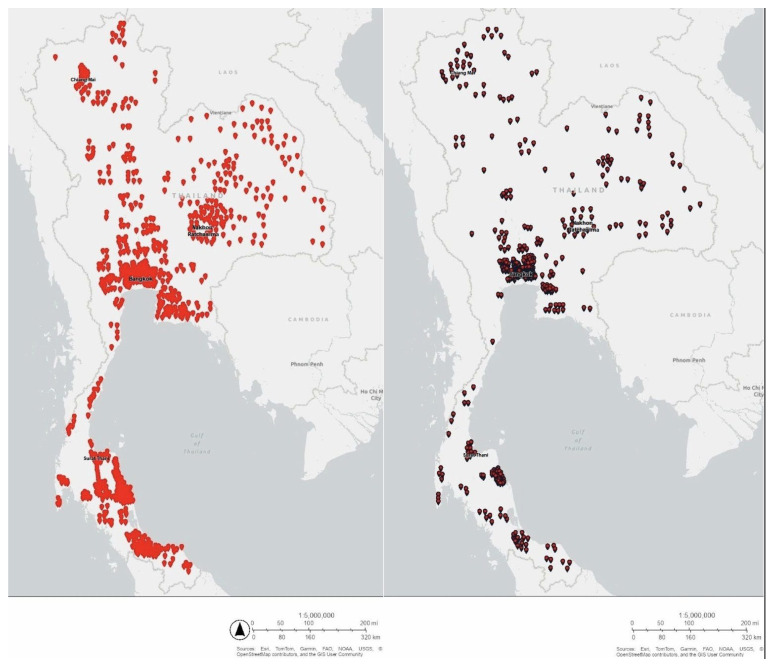
Spatial distribution of survey respondents who owned pet dogs (**Left**) and respondents who reported bite and/or scratch injuries caused by their own dogs (**Right**) across Thailand. The maps were generated using ArcGIS online version (ESRI Inc., Redlands, CA, USA); Each location pin represents one respondent.

The geographic distribution of dogs involved in bite and/or scratch incidents affecting their owners is presented in Figure 1. The central region reported the highest occurrence (21.56%, *n* = 227/479), followed by the southern (9.50%, *n* = 100/479) and northeastern (6.36%, *n* = 67/479) regions. Higher incidences were concentrated in major cities of each region, including Bangkok in the central region (22.55%, *n* = 108/479), Nakhon Si Thammarat province in the southern region (8.56%, *n* = 41/479), Nakhon Ratchasima province in the northeastern region (3.34%, *n* = 16/479), Chiang Mai province in the northern region (3.55%, *n* = 17/479), and Chonburi province in the eastern region (2.92%, *n* = 14/479).

### 3.3. Association Between Dog Biting and/or Scratching, and Owner-Related Factors

Non-binary owners reported a higher incidence of bites or scratches (50.79%, *n* = 32/63) compared to female (46.70%, *n* = 382/818) and male owners (37.79%, *n* = 65/172), although the difference was not statistically significant (*p* = 0.070). Owners with a postgraduate degree experienced biting and/or scratching by their dogs significantly (*p* = 0.004) more frequently (55.83%, *n* = 115/206) than those with a bachelor’s degree (43.47%, *n* = 263/605) or lower education levels (41.74%, *n* = 101/242). Region of residence was significantly associated with bite and/or scratch occurrence among dog owners (*p* = 0.003); 52.55% of the owners who live in the central part of Thailand had been injured by their dogs (*n* = 227/432), which was the highest percentage of all regions. The lowest occurrence was observed in the southern region (38.02%, *n* = 100/263) (Table 1).

### 3.4. Association Between Dog Biting and/or Scratching, and Dog-Related Factors

Dogs with chronic diseases were significantly (*p* = 0.022) more likely to be involved in bite and/or scratch incidents affecting their owners (50.77%, *n* = 198/390) compared with dogs without chronic disease (42.38%, *n* = 281/663) (Table 1). Vaccination routine was not significantly associated with bite and/or scratch occurrence. However, dogs that previously bit and/or scratched their owners were also significantly (*p* < 0.0001) more likely to bite and/or scratch strangers (32.02%, *n* = 161/447) than dogs that had never bitten or scratched their owners (19.50%, *n* = 102/523), with an odds ratio of 2.30 (95% CI: 1.73–3.10).

### 3.5. Common Owner–Dog Interactions That Triggered Dog Biting and/or Scratching

The interactions most commonly associated with bites or scratches among owners are detailed in Table 2, in which each dog owner could answer with more than one interaction. The leading triggers included causing discomfort during grooming (e.g., nail trimming, bathing, combing, or haircut) or administering oral medication (27.34%, *n* = 131/479); taking valuable objects such as food, toys, or bowls from the dog (24.42%, *n* = 117); interrupting dogs during fights (23.17%, *n* = 111/479); close physical contact including petting, hugging, or kissing (22.12%, *n* = 106/479); using bare hands to play or allowing dogs to chew or bite (21.29%, *n* = 102/479); and threatening the dog, for example by shouting or attempting to hit (18.78%, *n* = 90/479).

### 3.6. Common Stranger–Dog Interactions That Triggered Biting and/or Scratching

Biting and/or scratching of strangers by pet dogs was reported by 277 owners (26.3%). The principal interactions associated with these incidents were strangers entering the household area (70.4%, *n* = 195/277); approaching the dog’s territory (31.4%, *n* = 87/277); attempting to pet or touch the dog, including hugging or kissing (26.7%, *n* = 74/277); frightening the dog (22.0%, *n* = 61/277); and causing pain to the dog, such as hitting or stepping on it (10.1%, *n* = 28/277) (Table 2).

### 3.7. Biting and/or Scratching Triggered by Grooming and Giving Medication

Non-female owners reported a slightly higher occurrence of injuries during grooming or medication administration (32.99%, *n* = 32/97) compared to female owners (26.18%, *n* = 100/382), although the difference was not statistically significant (*p* = 0.18). Interestingly, a significant association was observed between owner age and the occurrence of bites or scratches during these interactions (*p* < 0.0001), with the highest occurrence among owners aged 18–30 years (40.67%, *n* = 61/150). Owners with an education level below a bachelor’s degree experienced a higher occurrence of bites or scratches in these contexts (32.67%, *n* = 33/101), but this difference was not statistically significant (*p* = 0.08). Similarly, owners with a single dog in the household also reported a higher occurrence (32.67%, *n* = 33/101), though this was not statistically significant (*p* = 0.09).

### 3.8. Biting and/or Scratching Triggered by Taking Food or Toys Away from Dogs

No significant differences were observed in the occurrence of bites or scratches triggered by removing food or toys from dogs across owner age (*p* = 0.85), gender (*p* = 0.72), education level (*p* = 0.20), or number of dogs in the household (*p* = 0.62).

### 3.9. Biting and/or Scratching Triggered by Close Physical Contact Including Petting, Hugging, or Kissing

No significant differences were observed in the occurrence of bites or scratches from close physical contact including petting, hugging, or kissing across owner gender (*p* = 0.67), age (*p* = 0.24), education level (*p* = 0.76), or numbers of dogs in the household (*p* = 0.26). Although not statistically significant, younger owners tended to experience bites or scratches from hugging and kissing more frequently than older owners, with the highest occurrence observed among those aged 18–30 years (27.33%, *n* = 41/150) and the lowest among owners aged > 50 years (16.67%, *n* = 14/84).

### 3.10. Knowledge of Dog Owners Toward Dog Bites and/or Scratches

The majority of dog owners (98.67%) reported having knowledge of rabies. However, 1.33% remained unaware of the seriousness of the disease, and 5.51% were uninformed about rabies prevention and the absence of effective treatment (Table 3). In addition, 19.85% of owners were unaware that microbial infections resulting from pet bites or scratches can cause severe illness or death. Furthermore, some owners lacked awareness of everyday canine behaviors that may pose a risk of injury, as detailed in Table 3. Notably, 37.80% of Thai dog owners reported lacking knowledge of enrichment strategies to reduce the occurrence of dog bite and scratch incidents (Table 3).

### 3.11. Attitudes of Dog Owners Toward Dog Bites and/or Scratches

Approximately one-third of owners perceived dog bites or scratches as normal behavior, while a smaller proportion (9.78%) believed that dog bites or scratches indicated the dog’s affection toward the owner. The majority of owners (71.60%) considered hugging and kissing their dogs to be an appropriate way to express love, and 71.51% endorsed the “alpha dog” concept, believing that the owner must assume the dominant role over the dog (Table 4).

### 3.12. Practices of Dog Owners Toward Dog Bites and/or Scratches

Subsequent to dog bite/scratch injuries, most of the dog owners (84.16%) performed appropriate first aid to the bite/scratch wounds by cleaning the wounds with clean water or saline solution, followed by soap, and/or applying an antiseptic; 14.48% of the owners cleaned the wounds with clean water or saline solution. Surprisingly, 1.36% did nothing to the wounds. The majority of dog owners (72.40%) visited a doctor after getting bitten or scratched by dogs, while some of the owners ignored it (27.60%). The reasons that made the dog owners go to the hospital were for a rabies and/or tetanus vaccination (69.38%) and for wound infection and/or suturing (21.87%). Unexpectedly, 8.75% of the dog owners did not consult a physician following being bitten and/or scratched by dogs.

## 4. Discussion

The present study demonstrated that the most frequent owner–dog interactions leading to bite or scratch injury were those causing discomfort during handling, such as grooming or the administration of medication. Such procedures can elicit aggression due to fear, anxiety, or pain, particularly in dogs with previous negative experiences [18,25]. Fearful or uncomfortable dogs may be more likely to display defensive or fear-related aggressive behaviors, which in some contexts can include biting or scratching compared to socially confident dogs [18,26]. Moreover, interactions such as taking valued items, such as toys or food, from dogs can also provoke bites or scratches in this study. This behavior, known as possessive aggression or resource guarding, is a commonly reported trigger of dog aggression in many studies [15,27]. Food and toy guarding is often linked to negative punishment strategies, such as when owners remove food or toys to penalize the dog [28].

Close physical contact, including petting, hugging, or kissing, was also commonly reported in association with bite and scratch incidents in the present study. Previous studies had suggested that some dogs may show behavioral signs of discomfort or avoidance during certain forms of close physical interaction, which could contribute to defensive responses in some contexts, included with children being particularly vulnerable to facial bites due to their height [29,30,31]. However, the present study did not directly evaluate canine emotional state, stress responses, or behavioral signaling during these interactions. Younger owners in the present study more frequently reported bite or scratch incidents associated with hugging and kissing their pets than older owners, as observed in both the present study and previous research [9]. Differences in interaction style may partly contribute to this pattern. The present study did not directly measure frequency or quality of physical interactions, and therefore, causal interpretations should be made cautiously. Moreover, biting and scratching injuries were more frequently reported among younger owners (18–30 years old), which may be attributed to limited experience and knowledge regarding dog care and behaviors.

In the present study, the primary cause of dogs biting strangers was entry into the house or dog area, a behavior commonly referred to as territorial aggression [16,20]. Additionally, territorial aggression may be associated with factors such as spoiled dogs, highly educated owners, and a preference for games like tug-of-war [20].

Many of the interactions associated with bite and scratch incidents in the present study are potentially modifiable through preventive education and humane handling strategies. Handling procedures such as grooming, restraint, and medication administration may become less stressful when dogs are gradually habituated and trained using reward-based approaches [18,28]. Positive reinforcement training, which encourages desired behaviors through rewards rather than punishment or confrontation, has been associated with improved welfare outcomes and reduced fear-related behavioral problems [28,32,33]. Such approaches may help owners manage daily care procedures more safely while preserving positive human–dog relationships.

Interestingly, dogs with chronic diseases were significantly more likely to bite or scratch their owners compared to healthy dogs. Although few studies have examined the association between physical illnesses and behavioral disorders, certain conditions, such as hyperandrogenism and chronic inflammatory diseases in dogs and cats, have been strongly linked to aggressive behavior [34]. Chronic inflammation in certain tissues, including the skin (dermatitis), oral cavity (stomatitis), urinary bladder (cystitis), and nasal cavity (rhinitis), can cause physical discomfort and pain which may in turn contribute to aggression-related behavior in dogs and cats [34].

Appropriate owner knowledge and attitudes are essential for preventing dog-related injuries. From the perspective of owners in the present study, many did not perceive dog bites or scratches as concerning, with some mistakenly interpreting these behaviors as expressions of affection. Moreover, the majority of owners regarded hugging and kissing their dogs as appropriate ways to demonstrate affection. Consistent with previous findings [32], such interactions may increase the risk of aggression, and bite or scratch injuries. These observations are supported by earlier studies showing that dogs subjected to hugging, kissing, or petting frequently exhibit signs of discomfort or anxiety, including gaze aversion, lip licking, panting, and attempts to withdraw, as well as defensive behaviors such as barking, growling, and biting [35,36]. Understanding canine communication is also essential for injury prevention. Dogs communicate through a range of behavioral signals that are highly dependent on social and environmental context [35,36]. Failure to recognize or appropriately respond to these signals may increase the likelihood of escalation during human–dog interactions. Consequently, owner education should not only focus on injury prevention but also on improving understanding of canine communication and promoting mutually positive interactions.

Many owners in the present study continued to endorse the “alpha dog” concept, believing that they must assert a dominant role over their pets. However, this traditional view is no longer supported by contemporary scientific evidence and is now regarded as an oversimplification and misinterpretation of canine social behavior [36]. Consequently, interactions involving verbal or physical threats towards dogs remain common, as observed in this study, and may provoke undesirable behaviors such as biting and scratching. This idea is not limited to Thailand; owner practices are still influenced by the “alpha dog” concept in many other countries [32,37]. Instead, relationship-based and reward-oriented methods that encourage learning, lessen fear, and increase animal welfare are the focus of behavioral medicine [28,33,38].

The fact that some dogs become more aggressive following sterilization is unknown to nearly half of the present study’s participants. The relationship between sterilization and canine aggression remains complex and may depend on behavioral motivation, individual temperament, age at sterilization, and environmental context. Although sterilization may reduce certain hormonally influenced behaviors, particularly reproductive and inter-male behaviors, evidence regarding aggression-related outcomes remains mixed [39,40]. Importantly, fear-related, anxiety-associated, or pain-related aggressive responses may not resolve solely through sterilization because these behaviors often arise from emotional state, learning history, or medical factors rather than reproductive hormones alone [39,40]. Consequently, sterilization should not be considered a universal strategy for preventing aggression-related behavior.

The present study demonstrated a high occurrence of bite injuries inflicted by owners’ own dogs (approximately 50%), although such incidents generally occurred less frequently than once every three months. This prevalence is considerably higher than that reported in emergency department-based data [11]. Our findings suggest that this discrepancy may be explained by the fact that many owners did not seek medical attention, or, if they did, did not present to emergency departments because the injuries were perceived as minor and because of limited awareness of the associated health risks. Of particular concern, 3.99% of owners reported that their dogs had never received rabies vaccination, which may increase public health risks associated with dog-related injuries. Rabies remains one of the most significant public health concerns in Thailand, with an estimated 47% of dogs at risk of rabies virus exposure [23]. Dogs account for the majority of reported animal cases, comprising approximately 90% of infections nationwide [41]. Notably, four provinces including Surin, Chiang Rai, Songkhla, and Chonburi have been identified as high-incidence areas for rabies [22], and these provinces were also among the locations reporting dog bite incidents in the present study, further underscoring the public health relevance of dog-related injuries in Thailand.

In the present study, bite and scratch injuries were combined in several analyses because the questionnaire was designed to evaluate dog-related injury experiences broadly and because both injury types share public health relevance, including risks of physical injury and zoonotic infection. These outcomes may reflect different behavioral processes. Biting is often interpreted as a deliberate oral defensive or aggressive response, whereas scratching may occur in broader contexts, including defensive movements, restraint avoidance, play, excitement, or incidental contact during handling. Because the present study relied on owner-reported experiences and did not directly evaluate canine behavioral motivation, body language, or contextual detail surrounding injury events, scratches should not be assumed to represent intentional aggression in all cases. Accordingly, the combined outcome used in this study should be interpreted as reflecting dog-related injury occurrence rather than a single underlying behavioral mechanism.

The present findings extend the previous dog-bite literature in several important ways. Many prior studies have relied on emergency department records or veterinary behavioral referrals, which predominantly capture severe injuries or clinically recognized aggression cases [11,15]. In contrast, the present survey assessed owner-reported bite and scratch injuries occurring within everyday household and social contexts. This broader approach allowed identification of interaction patterns, including grooming, medication administration, resource-related interactions, and close physical contact, that may not be consistently represented in clinical datasets. Furthermore, the integration of owner knowledge, attitudes, and practices provides insight into potentially modifiable behavioral and educational factors associated with injury occurrence.

Dog-related injuries remain an important public health concern; these findings should be interpreted within the broader context of promoting safe and positive human–dog relationships. Most dogs coexist safely with people, and injury risk is influenced by interaction context, owner knowledge, management practices, and environmental circumstances rather than representing a fixed characteristic of dogs themselves [42]. Educational programs that combine bite prevention with humane handling, positive reinforcement training, and improved recognition of canine communication may therefore benefit both human safety and animal welfare.

### Study Limitations

Several limitations should be acknowledged when interpreting the findings of the present study. First, the data were derived from owner-reported questionnaires and may therefore be subject to recall bias, subjective interpretation, and variability in respondents’ ability to recognize and accurately interpret subtle canine behavioral signals. Consequently, reporting accuracy may have varied among participants.

Second, respondent recruitment relied on voluntary online participation, which may have introduced demographic, socioeconomic, and self-selection biases. Although responses were collected from dog owners across Thailand, participation depended on internet accessibility and willingness to complete an online questionnaire. Therefore, certain populations may have been underrepresented, potentially affecting sample representativeness.

Third, female respondents constituted the majority of study participants. Although this demographic pattern may influence interpretation and generalizability, it is consistent with previous findings in human–animal interaction and companion animal research, where women frequently represent a disproportionately large proportion of survey respondents [8,9,43,44]. Nevertheless, the predominance of female participants may have influenced study outcomes if experiences, attitudes, or human–dog interaction practices differ according to respondent gender.

Fourth, this study did not directly assess canine emotional states, behavioral signaling, or injury severity during reported incidents. Furthermore, although bite and scratch injuries were combined in several analyses to represent dog-related injury occurrence, these outcomes may arise from distinct behavioral motivations and contextual circumstances. In particular, scratches may not necessarily reflect intentional aggression and may occur accidentally or during defensive, fearful, or excited movements. Accordingly, the findings should be interpreted as associations with owner-reported injury events rather than definitive evidence of specific behavioral mechanisms.

Finally, bite and scratch injuries were central outcomes of this study. The questionnaire did not employ highly standardized clinical or behavioral definitions regarding injury severity; skin penetration; or distinctions among biting, inhibited biting, and mouthing behaviors. Consequently, respondents may have interpreted and reported injury events differently, introducing potential heterogeneity in case classification and outcome reporting.

## 5. Conclusions

This study concludes that approximately half of surveyed dog owners in Thailand reported experiencing bite and/or scratch injuries from their own dogs and that approximately one-third of these dogs have also bitten and/or scratched strangers. Causing discomfort, particularly during grooming and medication administration, was identified as the main trigger for injuries caused by owned dogs, whereas entering into the household or territorial area was the primary trigger for injuries caused by unfamiliar dogs.

Furthermore, many dog owners in Thailand demonstrated gaps in knowledge regarding canine behavior, communication, and interactions associated with injury risk. These findings indicate an urgent need for targeted owner education to promote safer human–dog interactions and encourage context-sensitive approaches to injury prevention and bite-risk assessment.

## Figures and Tables

**Table 1:** Sociodemographic data and association of Thai dog owners who had been injured by their dog (biting and/or scratching) (N = 1053).

Characteristics	N (%)	Had Been Injured N (%)	Never Been Injured N (%)	*p*-Value
**Owner Gender**				0.070
Female	818 (77.69%)	382 (36.28%)	436 (41.40%)	
Male	172 (16.33%)	65 (6.179%)	107 (10.16%)	
Non-binary	63 (5.98%)	32 (3.04%)	31 (2.94%)	
**Owner age (year)**				0.626
18–30	347 (32.95%)	150 (14.25%)	197 (18.71%)	
31–40	271 (25.74%)	125 (11.87%)	149 (14.15%)	
41–50	269 (25.55%)	120 (11.40%)	146 (13.87%)	
>50	166 (15.76%)	84 (7.98%)	82 (7.79%)	
Owner Education *				0.004
Below bachelor’s degree	242 (22.98%)	101 (9.59%)	141 (13.39%)	
Bachelor’s degree	605 (57.46%)	263 (24.98%)	342 (32.48%)	
Postgraduate degree	206 (19.56%)	115 (10.92%)	91 (8.64%)	
Regions of Thailand *				0.003
Central Thailand	432 (41.03%)	227 (21.56%)	205 (19.47%)	
Northeastern	161 (15.29%)	67 (6.36%)	94 (8.93%)	
Eastern	71 (6.84%)	31 (2.96%)	41 (3.89%)	
Northern	125 (11.87%)	54 (5.13%)	71 (6.74%)	
Southern	263 (24.98%)	100 (9.50%)	163 (15.48%)	
**Number of dogs**				0.555
1 dog	435 (41.31%)	203 (19.28%)	232 (22.03%)	
2 dogs	256 (24.31%)	109 (10.35%)	147 (13.96%)	
≥3 dogs	362 (34.38%)	167 (15.86%)	195 (18.52%)	
Dogs with chronic diseases *				0.022
No chronic diseases	663 (62.96%)	281 (26.69%)	376 (35.71%)	
Had chronic disease	390 (37.04%)	198 (18.80%)	198 (18.80%)	
**Annual vaccination**				0.151
Regular vaccination	839 (79.67%)	391 (37.13%)	448 (42.55%)	
Non-regular vaccination or Never	214 (20.33%)	88 (8.36%)	126 (11.96%)	

* A *p*-value of ≤0.05 was considered statistically significant.

**Table 2:** Human–dog interactions that triggered dog biting and/or scratching. (The participants can choose more than one answer to this question.)

Owners (N = 479 Participants)	N (%)	Strangers (N = 277 Participants)	N (%)
Causing discomfort during grooming or administering oral medication	131 (27.34%)	Entering the house area	195 (70.40%)
Taking food or toys from the dog	117 (24.42%)	Getting into dog territory	87 (31.40%)
Interrupting dogs during fights	111 (23.17%)	Close physical contact including petting, hugging, or kissing	74 (26.70%)
Close physical contact including petting, hugging, or kissing	106 (22.12%)	Threatening the dog with voice or actions	61 (22.00%)
Using bare hands to play with dogs	102 (21.29%)	Causing pain, e.g., hit and step on	28 (10.10%)
Threatening the dog with voice or actions	90 (18.78%)	Taking food or toys from the dog	21 (7.60%)
When owners arrived or left the house	86 (18.00%)	Using bare hands to play with dogs	21 (7.60%)
Causing excitement, e.g., giving snacks, and playing time	84 (17.50%)	Causing excitement, e.g., giving snacks, and playing time	15 (5.40%)
Causing pain, e.g., hit and step on	68 (14.20%)	Interrupting dogs during fights	7 (2.50%)
Walking or running in the house area	48 (10.00%)	Causing discomfort during grooming or administering oral medication	13 (4.70%)
Unknown cause	33 (6.90%)	Unknown cause	17 (6.10%)
Getting into dog territory	31 (6.50%)	Interfering with pregnancy dog or their puppies	9 (3.20%)
Not paying attention to them	22 (4.60%)		

**Table 3:** Knowledge of dog owners toward dog bites and/or scratches (N = 1053).

Dog Owner Knowledge Questions	Answers	
	Known N (%)	Unknown N (%)
Animal saliva contains many types of pathogens that are dangerous to humans, causing illness or death.	996 (94.59%)	57 (5.41%)
Rabies can be transmitted through dog bites or scratches.	1039 (98.67%)	14 (1.33%)
Rabies can be fatal.	1039 (98.67%)	14 (1.33%)
There is no cure for rabies, but there is a vaccine to prevent the disease in both humans and animals.	995 (94.49%)	58 (5.51%)
Bacteria or fungi on your skin or from animals can enter a bite/scratch wound and cause you to become ill and even die.	943 (89.55%)	110 (10.45%)
Flesh-eating bacterial infections, and bacterial and fungal encephalitis can all be caused by pet bites or scratches.	844 (80.15%)	209 (19.85%)
If bitten or scratched by a dog, immediately wash the wound with soap and clean water and apply an antiseptic to prevent infection.	1034 (98.20%)	19 (1.80%)
You should see a doctor if you develop a fever or a pus-filled wound after a bite or scratch, as this indicates a bacterial infection.	1043 (99.05%)	10 (0.95%)
Wounds that are infected with bacteria must be treated with antibiotics.	974 (92.50%)	79 (7.50%)
If the dog that bit you has never been vaccinated against rabies, you should get a rabies vaccination.	1004 (95.35%)	49 (4.65%)
The tetanus vaccine is recommended after being bitten or scratched by an animal.	1004 (95.35%)	49 (4.65%)
Most dogs dislike being touched or approached by strangers.	1017 (96.58%)	36 (3.42%)
When a dog dislikes something or is stressed, they will turn their face away or try to escape.	922 (87.56%)	131 (12.44%)
Improper dog caring and training, such as being harsh, shouting, hitting, lacking time, or not getting enough exercise, can contribute to increased aggressive behavior in animals.	961 (91.26%)	92 (8.74%)
Raising and training dogs by rewarding appropriate behavior and ignoring inappropriate behavior will help them behave better, reduce stress, and decrease aggressive behavior.	855 (90.69%)	98 (9.31%)
Some dogs that are about to bite/scratch will exhibit threatening behaviors such as baring their teeth, puffing up their bodies.	1014 (96.30%)	39 (3.70%)
Some dogs that are going to bite or scratch will show no warning signs and will just attack.	924 (87.75%)	129 (12.25%)
Dogs that are sick or uncomfortable may become more aggressive.	855 (81.20%)	198 (18.80%)
In most cases, sterilization does not reduce aggression in dogs. In fact, some dogs become more aggressive after sterilization.	602 (57.17%)	451 (42.83%)
If you offer your hand or foot to a dog to chew on, they will think it is a toy and will develop a habit of biting your hands and feet.	758 (71.98%)	295 (28.02%)
Providing chewing/scratching toys, especially those with food compartments, can help reduce biting/scratching behavior towards the owner.	655 (62.20%)	398 (37.80%)
Aggressive behavior in dogs is often due to mental health issues that cannot be treated with training alone. It requires behavior modification efforts from both the owner and the pet.	702 (66.67%)	351 (33.33%)

**Table 4:** Attitudes of dog owners toward dog bites and/or scratches (N = 1053).

Dog Owner Attitude Questions	Answers	
	Yes N (%)	No N (%)
Do you think that dog bites/scratches are minor injuries and not life-threatening, and will heal on their own?	297 (28.21%)	756 (71.79%)
Do you think that if you are bitten or scratched by a dog, you need to get a rabies and/or tetanus shot?	1003 (95.25%)	50 (4.75%)
Do you think dogs that bark a lot usually do not bite?	181 (17.19%)	872 (82.81%)
Do you think dog bites or scratches are normal and natural behaviors of dogs?	331 (31.43%)	722 (68.57%)
Do you think that when a dog bites or scratches you, it is because it loves you?	103 (9.78%)	950 (90.22%)
Do you think hugging, kissing, and petting pets is the correct way to show love to animals?	754 (71.60%)	299 (28.40%)
Do you think owners need to act as the pack leader to make their pets obey and not harm them?	753 (71.51%)	300 (28.49%)

## Data Availability

The data presented in this study are available from the corresponding author upon reasonable request due to ethical reasons.

## Supplementary Materials

The following supporting information can be downloaded at https://www.mdpi.com/article/10.3390/ani16121809/s1, Table S1. National survey study of knowledge, attitude, and practice of dog owners toward dog bite and/or scratch injuries in Thailand.

## References

[1] Barroso C.S., Brown K.C., Laubach D., Souza M., Daugherty L.M., Dixson M. (2021). Cat and/or Dog Ownership, Cardiovascular Disease, and Obesity: A Systematic Review. Vet. Sci..

[2] Christian H., Wood L., Nathan A., Kawachi I., Houghton S., Martin K., McCune S. (2016). The association between dog walking, physical activity and owner’s perceptions of safety: Cross-sectional evidence from the US and Australia. BMC Public Health.

[3] Phillipou A., Tan E.J., Toh W.L., Van Rheenen T.E., Meyer D., Neill E., Sumner P.J., Rossell S.L. (2021). Pet ownership and mental health during COVID-19 lockdown. Aust. Vet. J..

[4] Hui Gan G.Z., Hill A.M., Yeung P., Keesing S., Netto J.A. (2019). Pet ownership and its influence on mental health in older adults. Aging Ment. Health.

[5] Powdrill-Wells N., Taylor S., Melfi V. (2021). Reducing Dog Relinquishment to Rescue Centres Due to Behaviour Problems: Identifying Cases to Target with an Advice Intervention at the Point of Relinquishment Request. Animals.

[6] Patronek G.J., Bradley J., Arps E. (2022). Saving normal: A new look at behavioral incompatibilities and dog relinquishment to shelters. J. Vet. Behav..

[7] Zapata I., Lilly M.L., Herron M.E., Serpell J.A., Alvarez C.E. (2022). Genetic testing of dogs predicts problem behaviors in clinical and nonclinical samples. BMC Genom..

[8] Boonhoh W., Wongtawan T., Sriphavatsarakom P., Waran N., Boonkaewwan C. (2023). The Validation of Thai version of Canine Behavioral Assessment and Research Questionnaire (C-BARQ) and the Exploration of Dog Ownership in Thailand. J. Vet. Behav..

[9] Boonhoh W., Wongtawan T., Sriphavatsarakom P., Waran N., Boonkaewwan C. (2023). Factors associated with pet dog behavior in Thailand. Vet. World.

[10] Sungkatavat P., Boonhoh W., Waran N., Wongtawan T. (2025). Prevalence of canine separation-related behaviors and associated factors in Thailand. J. Vet. Behav..

[11] Bhanganada K., Wilde H., Sakolsataydorn P., Oonsombat P. (1993). Dog-bite injuries at a Bangkok teaching hospital. Acta Trop..

[12] Amacher S.A., Søgaard K.K., Nkoulou C., Sutter R., Weisser M., Zingg S.S., Egli A., Hollinger A., Siegemund M. (2021). Bilateral acute renal cortical necrosis after a dog bite: Case report. BMC Infect. Dis..

[13] Selvi F., Stanbouly D., Stanbouly R., Baron M., Francois K., Halsey J., Marx R.E., Chuang S.-K. (2022). Early childhood (0 to 5 years) presents the greatest risk for facial dog bites. J. Oral Maxillofac. Surg..

[14] Westgarth C., Provazza S., Nicholas J., Gray V. (2024). Review of psychological effects of dog bites in children. BMJ Paediatr. Open.

[15] Notari L., Cannas S., di Sotto Y.A., Palestrini C. (2020). A Retrospective Analysis of Dog–Dog and Dog–Human Cases of Aggression in Northern Italy. Animals.

[16] Kleszcz A., Cholewińska P., Front G., Pacoń J., Bodkowski R., Janczak M., Dorobisz T. (2022). Review on Selected Aggression Causes and the Role of Neurocognitive Science in the Diagnosis. Animals.

[17] Blackwell E.J., Bradshaw J.W.S., Casey R.A. (2013). Fear responses to noises in domestic dogs: Prevalence, risk factors and co-occurrence with other fear related behaviour. Appl. Anim. Behav. Sci..

[18] Stellato A.C., Flint H.E., Dewey C.E., Widowski T.M., Niel L. (2021). Risk-factors associated with veterinary-related fear and aggression in owned domestic dogs. Appl. Anim. Behav. Sci..

[19] Tiira K., Lohi H. (2015). Early life experiences and exercise associated with canine anxieties. PLoS ONE.

[20] Pérez-Guisado J., Muñoz-Serrano A. (2009). Factors linked to territorial aggression in dogs. J. Anim. Vet. Adv..

[21] Rizk M.A., Abourizk N., Gadhiya K.P., Hansrivijit P., Goldman J.D. (2021). A Bite So Bad: Septic Shock Due to Capnocytophaga Canimorsus Following a Dog Bite. Cureus.

[22] Thichumpa W., Wiratsudakul A., Suwanpakdee S., Sararat C., Modchang C., Pan-ngum S., Prompoon N., Sagarasaeranee O., Premashthira S., Thanapongtharm W. (2024). Study of dog population dynamics and rabies awareness in Thailand using a school-based participatory research approach. Sci. Rep..

[23] Komol P., Sommanosak S., Jaroensrisuwat P., Wiratsudakul A., Leelahapongsathon K. (2020). The Spread of Rabies Among Dogs in Pranburi District, Thailand: A Metapopulation Modeling Approach. Front. Vet. Sci..

[24] Yamane T. (1967). Statistics: An Introductory Analysis.

[25] Ferreira M., Rodriguez M.A.P., Oliveira L.L.d.S., Maranhão C., de Oliveira N.J.F., Carvalho C.d.C.S., Afonso M.V.R., Madureira M.R. (2022). Stress in dogs during grooming in a pet shop. Rev. Bras. De Zootec..

[26] Eken Asp H., Fikse W.F., Nilsson K., Strandberg E. (2015). Breed differences in everyday behaviour of dogs. Appl. Anim. Behav. Sci..

[27] Bálint A., Rieger G., Miklósi A., Pongrácz P. (2017). Assessment of owner-directed aggressive behavioural tendencies of dogs in situations of possession and manipulation. R. Soc. Open Sci..

[28] Herron M.E., Shofer F.S., Reisner I.R. (2009). Survey of the use and outcome of confrontational and non-confrontational training methods in client-owned dogs showing undesired behaviors. Appl. Anim. Behav. Sci..

[29] Savalli C., Albuquerque N., Vasconcellos A.S., Ramos D., de Mello F.T., Serpell J.A. (2021). Characteristics associated with behavior problems in Brazilian dogs. Appl. Anim. Behav. Sci..

[30] Chen H.H., Neumeier A.T., Davies B.W., Durairaj V.D. (2013). Analysis of pediatric facial dog bites. Craniomaxillofac. Trauma Reconstr..

[31] Reisner I.R., Nance M.L., Zeller J.S., Houseknecht E.M., Kassam-Adams N., Wiebe D.J. (2011). Behavioural characteristics associated with dog bites to children presenting to an urban trauma centre. Inj. Prev..

[32] Howell T.J., Diverio S., Menor-Campos D.J. (2025). Beliefs About Cats and Dogs Among Pet Owners and Former Owners. Pets.

[33] Ziv G. (2017). The effects of using aversive training methods in dogs—A review. J. Vet. Behav..

[34] Boonhoh W., Poolkhet C., Waran N., Wongtawan T. (2025). Systematic review and meta-analysis of the occurrence and association of physical diseases and behavioural problems in dogs and cats. Appl. Anim. Behav. Sci..

[35] Sarrafchi A., David-Steel M., Pearce S.D., de Zwaan N., Merkies K. (2022). Effect of human-dog interaction on therapy dog stress during an on-campus student stress buster event. Appl. Anim. Behav. Sci..

[36] Walsh E.A., Meers L.L., Samuels W.E., Boonen D., Claus A., Duarte-Gan C., Stevens V., Contalbrigo L., Normando S. (2024). Human-dog communication: How body language and non-verbal cues are key to clarity in dog directed play, petting and hugging behaviour by humans. Appl. Anim. Behav. Sci..

[37] Menor-Campos D.J., Parreño M., Howell T., Diverio S. (2024). Exploring Myths and Misconceptions About Dog Behavior in a Spanish Population Sample. Anthrozoös.

[38] Westgarth C. (2016). Why nobody will ever agree about dominance in dogs. J. Vet. Behav..

[39] Farhoody P., Mallawaarachchi I., Tarwater P.M., Serpell J.A., Duffy D.L. (2018). Aggression toward familiar people, strangers, and conspecifics in gonadectomized and intact dogs. Front. Vet. Sci..

[40] McGreevy P.D., Wilson B., Starling M.J., Serpell J.A. (2018). Behavioural risks in male dogs with minimal lifetime exposure to gonadal hormones may complicate population-control benefits of desexing. PLoS ONE.

[41] Department of Livestock Development Thai Rabies Net. http://www.thairabies.net/trn/home/detailreport#.

[42] Hogue T.E., Howell H., Baslington-Davies A., Mills D.S. (2026). The Use of Structured Professional Judgement: A New Way to Understand and Assess Bite Risk from Dogs. Animals.

[43] Herzog H. (2021). Women Dominate Research on Human-Animal Bond. Psychology Today.

[44] Amiot C.E., Bastian B. (2015). Toward a psychology of human–animal relations. Psychol. Bull..

