# Supplementary material for: When Affection Becomes Risk: Human–Dog Interactions Associated with Bite and Scratch Injuries in a Survey of Dog Owners’ Knowledge, Attitudes, and Practices in Thailand
Source: Animals (Basel). 2026 Jun 11;16(12):1809. doi: 10.3390/ani16121809 (PMC13295716; doi:10.3390/ani16121809)
Supplement: Supplementary file 1 [file animals-16-01809-s001.zip › animals-4328878-supplementary.pdf]

Supplementary table S1 A National Survey Study of Knowledge, Attitude, and Practice of Dog Owners toward Dog Bite/Scratch Injuries in Thailand and Associated Factors

Questions	Answer choices
**Owner data**	
1. Gender	• Female<br>• Male<br>• Non-binary or N/A
2. Age range	• 18-30 year old<br>• 31-40 year old<br>• 41-50 year old<br>• 51-60 year old<br>• more than 60 year old
3. Education	• Lower than a bachelor degree<br>• Bachelor degree<br>• Higher than a bachelor degree
4. Which province do you live in?	• Amnat Charoen<br>• Ang Thong<br>• Ayutthaya<br>• Bangkok<br>• Bueng Kan<br>• Buriram<br>• Chachoengsao<br>• Chai Nat<br>• Chaiyaphum<br>• Chanthaburi<br>• Chiang Mai<br>• Chiang Rai<br>• Chonburi<br>• Chumphon<br>• Kalasin<br>• Kamphaeng Phet<br>• Kanchanaburi<br>• Khon Kaen<br>• Krabi<br>• Lampang<br>• Lanphun<br>• Loei<br>• Lopburi<br>• Mae Hong Son<br>• Maha Sarakham<br>• Mukdahan<br>• Nakhon Nayok<br>• Nakhon Pathom<br>• Nakhon Phanom<br>• Nakhon Ratchasima<br>• Nakhon Sawan

	• Nakhon SiThammarat<br>• Nan<br>• Narathiwat<br>• Nong Bua Lamphu<br>• Nong Khai<br>• Nonthaburi<br>• Pathum Thani<br>• Pattani<br>• Phang Nga<br>• Phatthalung<br>• Phayao<br>• Phetchabun<br>• Phetchaburi<br>• Phichit<br>• Phitsanulok<br>• Phrae<br>• Phuket<br>• Prachin Buri<br>• Prachuap Khiri Khan<br>• Ranong<br>• Roi Et<br>• Sa Kaeo<br>• Sakon Nakhon<br>• Samut Prakan<br>• Samut Sakhon<br>• Samut Songkhram<br>• Saraburi<br>• Satun<br>• Sing Buri<br>• Sisaket<br>• Songkhla<br>• Sukhothai<br>• Suphan Buri<br>• Surat Thani<br>• Surin<br>• Tak<br>• Trang<br>• Trat<br>• Ubon Thani<br>• Uttaradit<br>• Yala<br>• Yasothon
**Dog data**	
5. How many dogs do you have?	• None<br>• 1 dog<br>• 2 dogs<br>• 3 dogs or more
6. Do(es) your dog(s) receive a rabies vaccine annually?	• Every dog receives rabies vaccines.<br>• Some dogs receive rabies vaccines.<br>• Dogs discontinuously receive rabies

	vaccines.<br>● Never done
7. Do(es) your dog(s) have any disease?	● None<br>● Skin disease<br>● Heart disease<br>● Renal or urinary disease<br>● Arthritis or musculitis<br>● Paraphegia or paralysis<br>● Liver disease<br>● Gastrointestinal disorders eg. diarrhea, constipation<br>● Blood parasite infection<br>● Cancer<br>● Others please define ……………….
**Dog aggressiveness towards owners**	
8. Have you and/or your family members ever been harmed by your dog?	● Never<br>● Yes, I have been bitten.<br>● Yes, I have been scratched.<br>● Yes, I have been bitten and scratched.
9. How often have you and/or your family members been bitten/scratched by your dog(s)?	● Everyday<br>● Every week<br>● Every month<br>● Every 3 months or more
10. The reasons of your dog(s) biting/scratching you and/or your family members (You can choose more than 1 reason.)	● Possessive of food or toys<br>● Causing fear or frighten eg. punish, scold, threaten etc.<br>● Causing pain eg. hit, step on etc.<br>● Interfering during dogs fighting<br>● Interfering pregnancy dog or their puppies<br>● Play with bare hands<br>● Skinship eg. hug, kiss, pet, touch etc.<br>● When owner get out/in the house<br>● Walk or run in the house area<br>● Get in their territory<br>● Doing something that dogs dislike eg. grooming, bathing etc.<br>● Hold other dog or kid<br>● Not pay attention to them<br>● Stimulate excitement eg. snack, play time etc.<br>● Unknown cause<br>● Others please define ……………….

**Dog aggressiveness towards strangers**	
11. Have your dogs ever bitten/scratched strangers?	• Never<br>• Yes, they have bitten/scratched adult strangers.<br>• Yes, they have bitten/scratched child strangers.<br>• Yes, they have bitten/scratched either adult or child strangers.
12. The reasons of your dog(s) biting/scratching strangers (You can choose more than 1 reason.)	• Possessive of food or toys<br>• Causing fear or frighten eg. punish, scold, threaten etc.<br>• Causing pain eg. hit, step on etc.<br>• Interfering during dogs fighting<br>• Interfering pregnancy dog or their puppies<br>• Play with bare hands<br>• Skinship eg. hug, kiss, pet, touch etc.<br>• When owner get out/in the house<br>• Walk or run in the house area<br>• Get in their territory<br>• Doing something that dogs dislike eg. grooming, bathing etc.<br>• Hold other dog or kid<br>• Not pay attention to them<br>• Stimulate excitement eg. snack, play time etc.<br>• Unknown cause<br>• Others please define ………………
**Attitude of dog owners**	
13. Do you think that dog bites/scratches are minor injuries, not life-threatening, and will heal on their own?	• Yes<br>• No
14. Do you think that if you are bitten or scratched by a dog, you need to get a rabies and/or tetanus shot?	• Yes<br>• No
15. Do you think dogs that bark a lot usually don't bite?	• Yes<br>• No
16. Do you think dog bites or scratches are normal and natural behavior of dogs?	• Yes<br>• No

17. Do you think that when a dog bites or scratches you, it's because it loves you?	• Yes<br>• No
18. Do you think hugging, kissing, and petting pets is the correct way to show love to animals?	• Yes<br>• No
19. Do you think owners need to act as the pack leader to make their pets obey and not harm them?	• Yes<br>• No
**Dog owner Knowledge**	
20. Animal saliva contains many types of pathogens that are dangerous to humans, causing illness or death.	• Known<br>• Unknown
21. Rabies can be transmitted through dog bites or scratches.	• Known<br>• Unknown
22. Rabies can be fatal.	• Known<br>• Unknown
23. There is no cure for rabies, but there is a vaccine to prevent the disease in both humans and animals.	• Known<br>• Unknown
24. Bacteria or fungi on your skin or from animals can enter a bite/scratch wound and cause you to become ill and even die.	• Known<br>• Unknown
25. Flesh-eating bacterial infections, bacterial and fungal encephalitis can all be caused by pet bites or scratches.	• Known<br>• Unknown
26. If bitten or scratched by a dog, immediately wash the wound with soap and clean water and apply an antiseptic to prevent infection.	• Known<br>• Unknown
27. You should see a doctor if you develop a fever or a pus-filled wound after a bite or scratch, as this indicates a bacterial infection.	• Known<br>• Unknown

28. Wounds that are infected with bacteria must be treated with antibiotics.	• Known<br>• Unknown
29. If the dog that bit you has never been vaccinated against rabies, you should get a rabies vaccination.	• Known<br>• Unknown
29. The tetanus vaccine is recommended after being bitten or scratched by an animal.	• Known<br>• Unknown
30. Most dogs dislike being touched or approached by strangers.<br>31. When a dog dislikes something or is stressed, they will turn their face away or try to escape.	• Known<br>• Unknown
32. Improper dog upbringing and training, such as being harsh, shouting, hitting, lacking time, or not getting enough exercise, can contribute to increased aggressive behavior in animals.	• Known<br>• Unknown
33. Raising and training dogs by rewarding appropriate behavior and ignoring inappropriate behavior will help them behave better, reduce stress, and decrease aggressive behavior.	• Known<br>• Unknown
34. Some dogs that are about to bite/scratch will exhibit threatening behaviors such as baring their teeth, puffing up their bodies.	• Known<br>• Unknown
35. Some dogs that are going to bite or scratch will show no warning signs and will just attack.	• Known<br>• Unknown
36. Dogs that are sick or	• Known

uncomfortable may become more aggressive.	• Unknown
37. In most cases, sterilization does not reduce aggression in dogs. In fact, many dogs become more aggressive after sterilization.	• Known<br>• Unknown
38. If you offer your hand or foot to a dog to chew on, they will think it's a toy and will develop a habit of biting your hands and feet.	• Known<br>• Unknown
39. Providing chew/scratching toys, especially those with food compartments, can help reduce biting/scratching behavior towards the owner.	• Known<br>• Unknown
40. Aggressive behavior in dogs is often due to mental health issues that cannot be treated with training alone. It requires behavior modification efforts from both the owner and the pet.	• Known<br>• Unknown
**Dog owner practice**	
41. When you were bitten or scratched by a dog, did you see a doctor?	• Yes<br>• No
42. When you are bitten or scratched by a dog, what do you do to the wound?	• Clean the wound with clean water or saline solution, followed by soap, and/or apply an antiseptic.<br>• Clean the wound with clean water or saline solution.<br>• Do nothing
43. If you are bitten or scratched by a dog, what reasons would you go to see a doctor?	• For a rabies and/or tetanus vaccination?<br>• Wound infection and suturing<br>• Did not see a doctor
